# The Differential Diagnosis of Intestinal Hæmorrhage

**Published:** 1912-11-09

**Authors:** 


### THE DIFFERENTIAL DIAGNOSIS OF INTESTINAL HÆMORRHAGE.

### Some Symptoms of Obstruction and Disease.

The passage of blood in the stools in certain cases is the most prominent sign of disease, and the symptom for which the patient seeks advice. The blood may vary in appearance and quantity. It may be bright red, dark red, purplish, or black like tar. The quantity may vary from a few streaks adhering to the fæces to a pint or more of almost pure blood. In order to be in a position to diagnose the cause and the source of the hæmorrhage, it is of the utmost importance to observe the amount and character of the blood, to note the presence or absence of mucus, mucous casts, shreds of mucous membrane, or sloughs, and to notice the condition of the fæces, whether formed or loose, whether pale or of natural colour. When the motions contain black, tarry-looking blood the condition is termed melæna, and this indicates that the source of the hæmorrhage is high in the alimentary tract where the blood has come into contact with gastric juice. The black appearance is due to the action of this digestive fluid converting the hæmoglobin into hæmatin. If the hæmorrhage is from the duodenum, and it is free, a mixture of black and bright red blood may be found; if from the ileum the blood may be mixed intimately with the fæces; if from the colon, it is less well mixed; and when from the lower part of the colon or rectum, may be found independently of any fæcal material.

### Foreign Bodies.

The impaction of a foreign body, such as a pin, nail, or sharp piece of bone, in the mucous membrane of the gut, especially of the rectum, may lead to ulceration and hæmorrhage. A man who was suffering from pain in the rectum, which was of an excruciating character when he passed a motion, and who for some time had noticed a varying amount of blood with each evacuation, was found to have a sharp piece of rabbit-bone impacted in the wall of his rectum about an inch and a half above his anus. The removal of this was followed by complete relief of his symptoms. In such cases a careful rectal examination is imperative in order to arrive at a correct diagnosis.

### Trauma.

Hæmorrhage from the rectum may follow injuries to the abdomen, and indicates some breach of continuity of the mucous membrane of the intestine. The injury may be direct—*e.g.* a stab or gunshot wound—or indirect when there is no external lesion, but the gut has been injured or ruptured as the result of a violent crushing blow. The diagnosis of hæmorrhage from such a cause presents no difficulty, as the injury is usually of such a nature as to obtrude itself as the origin of the bleeding. The nozzle of an enema tube has excoriated the mucosa before now and led to the passage of blood per anum.

### Intussusception.

The passage of bright-red blood and mucus per rectum is the most characteristic sign of this disease and occurs in more than 80 per cent. of the acute cases. If the stool has not been kept for inspection a digital examination of the rectum will reveal the presence of blood and mucus in the lower bowel.

Acute intussusception is by far the commonest form of acute intestinal obstruction in infants and children. The onset is sudden, commencing with severe griping pains in the abdomen, the child crying out with pain and drawing up his legs. Pain is followed by nausea, vomiting, tenesmus, the passage of thin fæces and blood and then of blood and mucus. The abdomen may or may not be distended. A cylindrical or sausage-shaped tumour may be felt in the abdominal cavity, its position depending on the situation and size of the intussusception. If seen early the tumour is usually detected in the right loin, later it may be felt lying across the abdomen just above the level of the umbilicus, in the splenic region, in the left loin, in the right iliac region, in the rectum, or may be seen even to project from the anus.

It is most liable to be mistaken for dysenteric diarrhœa or enteritis, in which condition there is diarrhœa with the passage of blood and mucus, but there is no tumour, and the blood is mixed, not only at the beginning but throughout the course of the disease, with thin fæcal matter.

A rectal examination is of the utmost importance in all cases of hæmorrhage from the rectum in children, for it may not only reveal the presence of blood and mucus in the lower bowel, but the entering portion of an intussusception may be felt in the rectum, or by means of a finger in the rectum and the other hand on the abdomen the tumour may be detected and its characteristic features made out.

The passage of blood and mucus is also one of the most important signs of chronic intussusception, a condition which affects adults and is usually the result of carcinoma of the colon.

### Dysentery.

The disease is an acute inflammation of the large intestine, which results in extensive ulceration of the mucous membrane.

It usually commences with diarrhœa, five or six loose brownish-yellow motions being passed during the first two or three days, then as many as from ten to sixty evacuations may occur in the twenty-four hours. At this stage the stools are scanty, and consist almost entirely of blood, mucus, serum, and pus, with shreds of necrosed mucous membrane. In the later stages pure blood may be passed, or blood may appear in clots floating in a red serous fluid.

Associated with the diarrhœa and the passage of blood and mucus there are severe griping abdominal pains, tenesmus, burning pain in the anus and rectum, and frequent micturition. The patient loses flesh, becomes anæmic, the tongue is furred, the breath offensive, and the temperature rises to 101° F. or 102° F.

There are two distinct varieties of dysentery:

(*a*) acute specific dysentery; (*b*) amœbic dysentery. The former is prevalent in Japan, and a specific bacillus was found by Shiga to be the cause of it. This same micro-organism was found by Flexner, who investigated the cause of dysentery in the Philippine Islands, and the same bacillus has been discovered in the epidemic ulcerative colitis or asylum dysentery, which occurs in lunatic asylums in this country. The bacillus dysenteriæ (Shiga) is about the same size as the bacillus typhosus, and slightly motile. It grows readily on all culture media, does not liquefy gelatine, does not coagulate milk, does not ferment sugar, and renders litmus milk alkaline after first of all making it slightly acid. The blood serum of a patient suffering from this disease agglutinates the bacillus dysenteriæ of Shiga. The isolation of this bacillus from the stools and a positive agglutination reaction would confirm the diagnosis.

The amœbic form occurs in Egypt, India, America, and many parts of the tropics. The diagnosis can be made by finding the amœbæ in the stools in the little flakes of mucus which are passed with the blood. The amœba of dysentery measures about 15 to 20 $\mu$ in diameter, and consists of an inner granular zone containing a nucleus and one or two vacuoles and an outer clear zone. Amœbæ may also be found containing red blood corpuscles. In some cases they are present in enormous numbers.

### Colitis.

Simple disease is a catarrhal inflammation of the mucous membrane of the large intestine without ulceration. Diarrhœa is the chief symptom, and it usually comes on suddenly. Many stools may be passed in the twenty-four hours, consisting mainly of fluid blood and mucus for the first few days, and then of more fæcal matter and less blood and mucus during the next week or two. There is paroxysmal abdominal pain along the course of the colon and tenderness over the sigmoid flexure, but no tenesmus as in dysentery. The temperature may rise to 101° F. or 102° F., and the tongue is covered with a white fur. This disease may be primary, or may occur as a complication of chronic Bright's disease, pneumonia, or septicæmia.

In membranous colitis, blood and membranous-looking mucous casts, which vary in length from 1 or 2 to 12 or more inches, are passed in the stools. In contrast to the other forms of colitis, constipation is present rather than diarrhœa. The chief symptoms are abdominal pain, nausea, flatulence, and pain on defæcation. The patients are mainly unmarried women of nervous temperament.

In ulcerative colitis there may be only a few ulcers in the large intestine or, on the other hand, the ulceration may be so extensive that only a few small areas of mucous membrane remain. In some cases there are a number of islets of mucous membrane, the edges of which are undermined so that a curious polypoid appearance is produced. The ulcers are very irregular both in shape and size, and stretching between and across them may be seen thin bands of mucous membrane which are completely undermined. The muscular coat of the intestine is exposed in the floor of the ulcers.

Diarrhœa and the passage of blood and mucus in the stool are the most prominent symptoms. As many as ten or twelve dark fluid, foul-smelling motions containing blood, pus, and shreds of mucous membrane may be passed in the twenty-four hours. The blood may be liquid, or, if in large amounts, it appears in the form of clots.

There is severe griping abdominal pain, which becomes worse when the bowels are opened, but tenesmus is rare. In addition, the patient complains of nausea, vomiting, thirst, and profuse sweating. The abdomen becomes distended and tympanitic, the temperature rises from 101° F. to 103° F., the pulse rate becomes rapid, and very soon the patient becomes anæmic, wasted, and exhausted. A rectal examination should be made, as in some cases ulceration can be felt, or may be seen by means of a speculum.

### Tuberculous Ulceration.

Hæmorrhage from tuberculous ulceration of the intestine is a rare and unusual symptom, considering the frequency of the lesion. The lower part of the ileum is most commonly affected, but the cæcum and colon may also be involved. The ulcers are irregular in shape and in the transverse axis of the bowel, the edges are thickened, the submucous and muscular coats are involved, and both on the mucous and the peritoneal surfaces yellow tubercles may be seen, those on the mucous side being caseous and breaking down. Hæmorrhage occurs from erosion of blood-vessels.

Tuberculous ulceration may be primary or secondary. Primary ulceration is frequently associated with caseation of the mesenteric glands, and this is the form which occurs in children. The chief symptoms are diarrhœa, emaciation, weakness, pyrexia, anæmia, distended abdomen, the presence of irregular-shaped tumours in the abdomen which are due to enlarged glands, thickening of the omentum or matting together of the intestines, and the association of such signs and symptoms with the occurrence of blood in the motions would justify a diagnosis of tuberculous ulceration of the intestine.

### Its Rate Per Cent.

In adults intestinal ulceration is nearly always secondary to pulmonary tuberculosis, and is present in more than 50 per cent. of the fatal cases. If pulmonary tuberculosis is complicated by tuberculous laryngitis, ulceration of the intestine is present in more than 90 per cent. of the cases. Hæmorrhage from the bowel, therefore, occurring in a case of pulmonary tuberculosis would point to tuberculous ulceration of the intestine as its most probable cause, but in the majority of the patients there are neither signs nor symptoms of the intestinal lesions, though post-mortem examination reveals scores of the tuberculous ulcers.

(*To be continued.*)

